# Characterization of DREB family genes in *Lotus japonicus* and *LjDREB2B* overexpression increased drought tolerance in transgenic *Arabidopsis*

**DOI:** 10.1186/s12870-024-05225-y

**Published:** 2024-06-04

**Authors:** Dan Wang, Yuanyuan Zeng, Xiuxiu Yang, Shuming Nie

**Affiliations:** Key Laboratory of Southwest China Wildlife Resources Conservation (Ministry of Education), College of Life Science, China West Normal University, Nanchong, 637009 China

**Keywords:** DREB, *Lotus japonicus*, Transcription factor, *LjDREB2B*, Drought tolerance

## Abstract

**Background:**

Drought stress affects plant growth and development. DREB proteins play important roles in modulating plant growth, development, and stress responses, particularly under drought stress. To study the function of DREB transcription factors (TFs), we screened key DREB-regulating TFs for drought in *Lotus japonicus*.

**Results:**

Forty-two DREB TFs were identified, and phylogenetic analysis of proteins from *L. japonicus* classified them into five subfamilies (A1, A2, A4, A5, A6). The gene motif composition of the proteins is conserved within the same subfamily. Based on the cis-acting regulatory element analysis, we identified many growth-, hormone-, and stress-responsive elements within the promoter regions of DREB. We further analyzed the expression pattern of four genes in the A2 subfamily in response to drought stress. We found that the expression of most of the LjDREB A2 subfamily genes, especially *LjDREB2B*, was induced by drought stress. We further generated *LjDREB2B* overexpression transgenic *Arabidopsis* plants. Under drought stress, the growth of wild-type (WT) and overexpressing *LjDREB2B* (OE) *Arabidopsis* lines was inhibited; however, OE plants showed better growth. The malondialdehyde content of *LjDREB2B* overexpressing lines was lower than that of the WT plants, whereas the proline content and antioxidant enzyme activities in the OE lines were significantly higher than those in the WT plants. Furthermore, after drought stress, the expression levels of *AtP5CS1*, *AtP5CS2*, *AtRD29A*, and *AtRD29B* in the OE lines were significantly higher than those in the WT plants.

**Conclusions:**

Our results facilitate further functional analysis of *L. japonicus DREB*. *LjDREB2B* overexpression improves drought tolerance in transgenic *Arabidopsis*. These results indicate that DREB holds great potential for the genetic improvement of drought tolerance in *L. japonicus*.

**Supplementary Information:**

The online version contains supplementary material available at 10.1186/s12870-024-05225-y.

## Background

Drought influences plant growth and development. This seriously affects the efficiency of agricultural production, and ecological and environmental security. Plants form a complex transcriptional regulatory network that resists drought stress [1]. Transcription factors (TFs) play an important role in drought stress response. They specifically bind to cis-acting elements in promoter regions and synergistically regulate the expression of downstream genes [2]. Dehydration-responsive element binding (DREB) family TFs are a subfamily of the APETALA2/Ethylene-Responsive Element Binding Factor (AP2/ERF) which regulates the response to biotic and abiotic stress [3, 4]. Recent studies demonstrated that DREB TFs respond to drought, salt, cold, heat, and other abiotic stressors in plants [5, 6].

DREB genes play crucial roles in plant hormone signaling pathways for protection against pathogens and abiotic stress [7]. The response of plants to stress is controlled by multiple genes in the signal transduction network, and DREB participates in multiple signal transduction pathways [8]. Adversity can induce the expression of DREB genes and regulate the expression of target genes in response to stress [9, 10]. DREB specifically binds to the dehydration response element/C-repeat (DRE/CRT) and cis-acting elements (G/ACCGAC) in the promoter regions of stress resistance genes [11]. DREB interacts with ABA-responsive element-binding proteins (AREB) and heat shock transcription factors (HSF) in response to adversity and regulates stress resistance [12].

The DREB subfamily can be divided into six subgroups, namely A1–A6, in *Arabidopsis* [13]. The DREB A1 subfamily members are sensitive to cold stress and regulate the expression of cold stress-related genes [14]. The expression of DREB 2 A is induced by drought and overexpression of DREB A2 significantly improved drought stress tolerance in transgenic *Arabidopsis* [15]. *Arabidopsis AtTINY* belongs to the DREB A3 family Overexpression of *AtTINY* improves cold resistance by upregulating cold stress-related genes such as *COR6.6*, *COR15A*, and *COR78* [16]. *SlDREBA4* of tomato DREB A4 family is involved in regulating heat resistance [17]. *GmDREB2* was classified into the A5 subgroup; *GmDREB2* binds to the DRE element and overexpression of *GmDREB2* improves drought resistance under drought stress [18]. The DREB A6 gene *CiDREB6* improves the heat tolerance of plants by regulating the expression of several heat shock protein (Hsp) genes [19]. The mechanism by which DREB participates in stress regulation is complex and different species have different stress resistance mechanisms.

The DREB family of genes plays an important role in the drought stress [20]. The sequences and expression patterns of DREB genes have been identified and analyzed from model plants to higher plants [21, 22]. For example, using comprehensive genome-wide screening, 20 *AcoDREB* genes in pineapple (*Ananas comosus*) were identified, some of the *AcoDREB* genes responded to drought stress [23]. The expression of FvDREB8 in subgroup A2 is rapidly induced under drought stress [24]. DREB TFs RAP2.4 activates cuticular wax biosynthesis in transgenic *Arabidopsis* leaves under drought stress [25]. Overexpression of *OsDREB* improved drought resistance in rice [26]. Overexpression of *StDREB2* enhanced drought stress tolerance in cotton [27]. Overexpression of *DcDREB1A* enhanced drought tolerance in transgenic *Arabidopsis* and modulated lignin levels by regulating lignin-biosynthesis-related genes [28]. These studies show that the DREB family genes play an important role in plant resistance to drought stress.

*Lotus japonicus* is an important leguminous forage that is used as a protein feed source, biological nitrogen fixation resource, and in ecological conservation. Drought significantly affects the growth and geographical distribution of *L. japonicus*. Therefore, it is important to breed *L. japonicus* cultivars with enhanced drought resistance. Expression of *Medicago truncatula MtDREB2A* is induced by drought, salt, and heat stress, and overexpression of *MtDREB2A* results in significant dwarfism in transgenic *M. truncatula* plants [29]. Mizoi found that *GmDREB2A* was induced by drought, heat, and low-temperature stress and improved drought resistance in transgenic *Arabidopsis* [30]. The DREB family gene in the legumes has been identified and studied, while the DREB in *L. japonicus* has not been studied. In the present study, we identified 42 DREB TFs in *L. japonicus*. To provide insights into the evolution and function of DREB genes, we analyzed their phylogeny, physicochemical properties, structure, classification, and promoter cis-acting elements. We found that *LjDRE2B* expression was strongly induced by drought stress. Further investigation showed that overexpression of *LjDRE2B* increased the drought resistance of transgenic *Arabidopsis* plants.

## Results

### Phylogenetic analysis of the DREB gene family

**Fig. 1:**
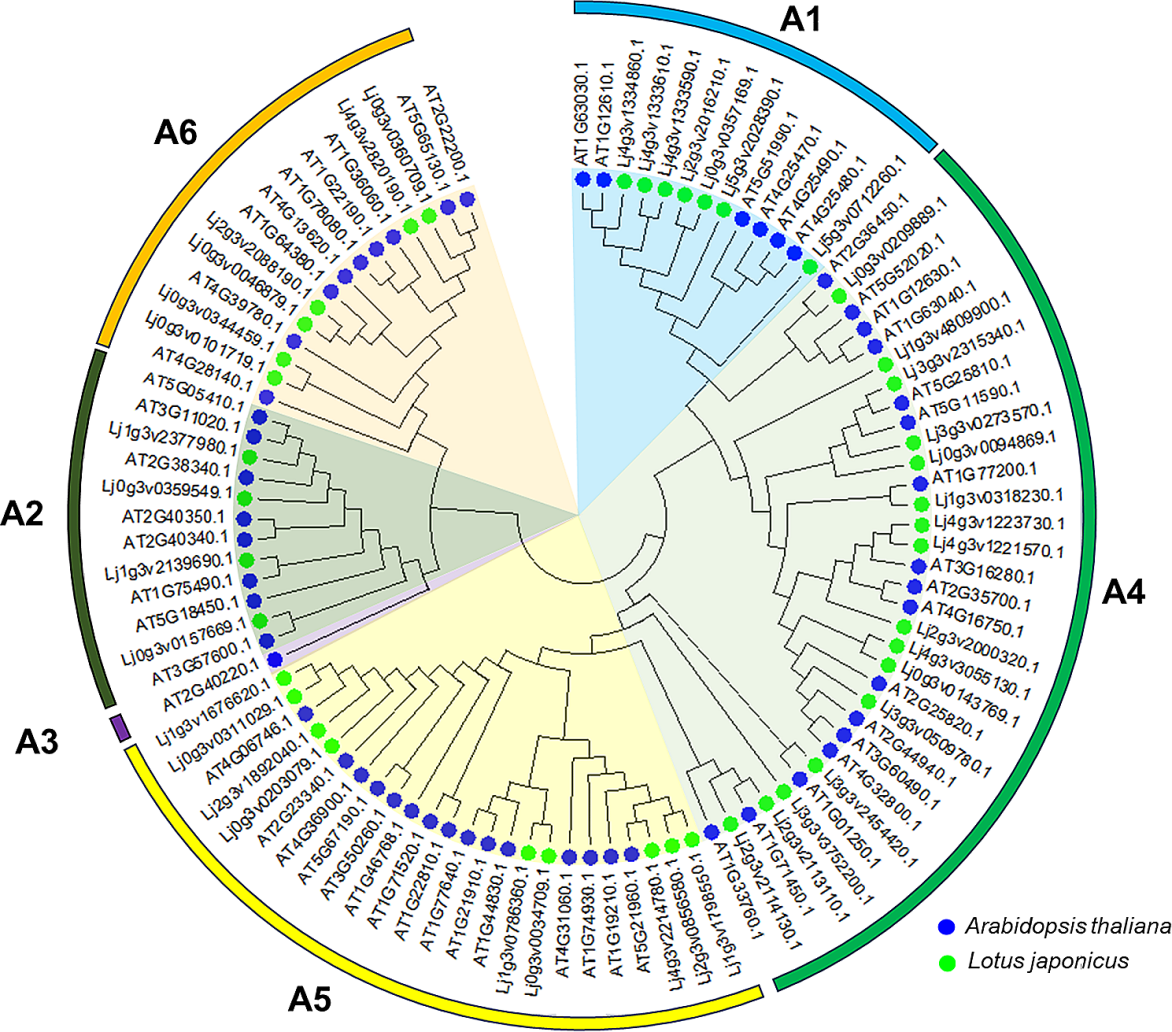
Phylogenetic tree of DREB proteins from *Lotus japonicus* and *Arabidopsis thaliana*. The tree was divided into six clades, which are marked by different colors and named as A1, A2, A3, A4, A5, and A6. The tree was constructed using the neighbor-joining algorithm with 1000 bootstraps based on the amino acid sequence of DREB proteins

A phylogenetic tree was constructed using the full-length DREB protein sequences of *Lotus japonicus* and *Arabidopsis* and was divided into six groups, A1 to A6 (Fig. 1). *Lotus japonicus* and *M. truncatula* were located on branches close together and exhibited a close kinship relationship. *Glycine max* had more family members, which is closely related to their chromosome numbers (Fig. S1A). The proportions of subgroups among different species were similar (Fig. S1B). The number of DREB genes in group A4 was the highest, ranging from 27.27 to 38.64%, whereas group A3 had the lowest, ranging from 1.41 to 2.27%.

### Physicochemical properties analysis of the DREB gene family

According to the sequence analysis of the six species, amino acid lengths ranged from 83 to 627 amino acids (aa), with molecular weights varying from 9408.36 Da to 60,208.07 Da (Table S1). *Lotus japonicus* showed the minimum variation in length (83–526 aa), whereas *Trifolium pratense* (106–627 aa) showed the maximum variation in length. The isoelectric points (pI) ranged from 3.8867 to 12.1988, indicating members from different physiological environments. More than 80.07% of the DREB family proteins from the six species were weakly acidic because their pI was < 7. The grand average of hydropathicity (GRAVY) values was negative and ranged from − 1.186 to -0.279, thus implying their hydrophilic nature. The aliphatic indices ranged from 48.19 to 80.73.

### Conservative domain and motif composition analysis

**Fig. 2:**
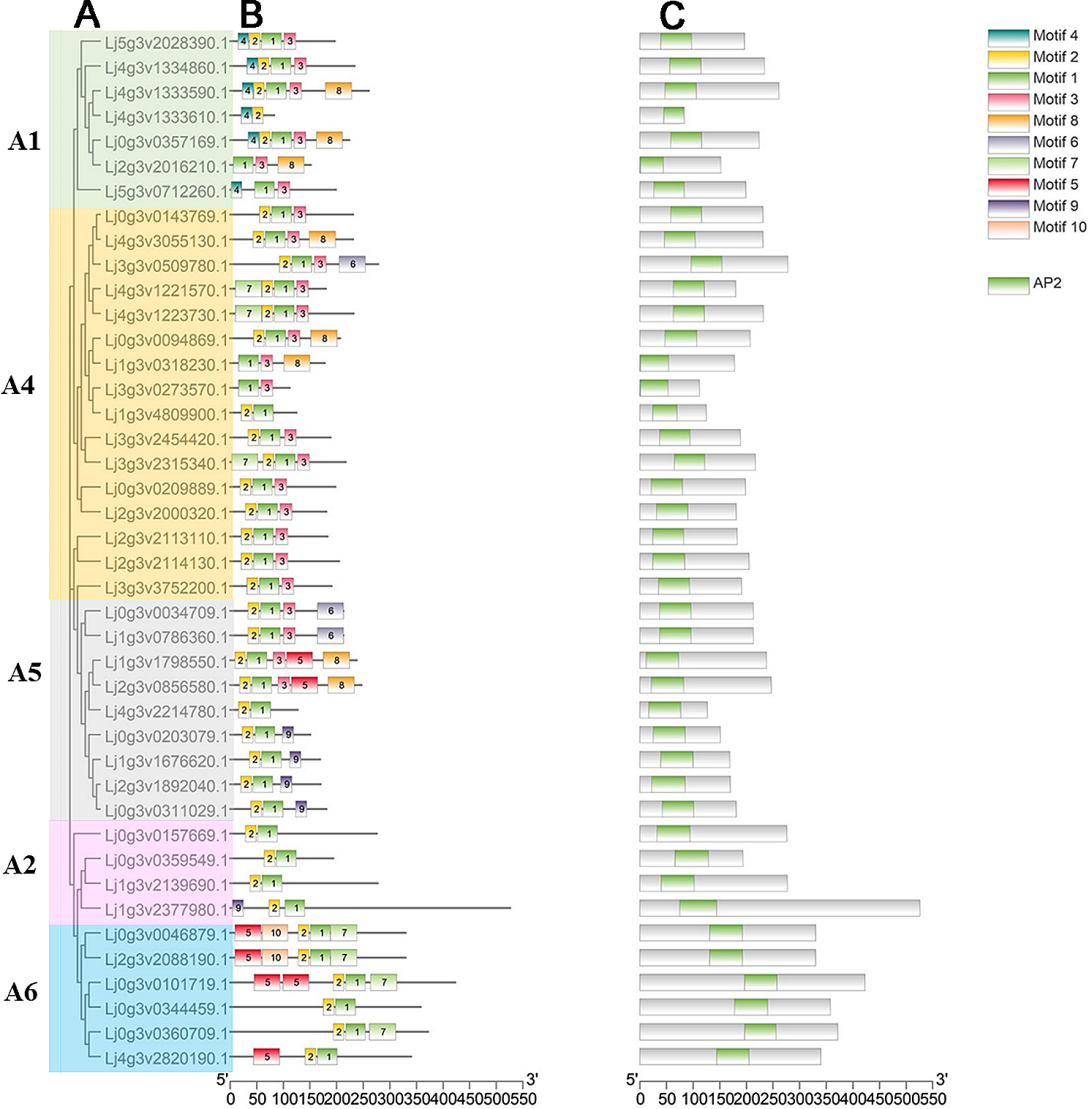
Phylogenetic relationships and conserved motifs of DREB genes from *Lotus japonicus*. **A** Phylogenetic tree of DREB proteins of *Lotus japonicus*. **B** Conserved motif of DREB proteins analyzed by MEME suite. Different colored boxes indicate different motif types. **C** Conserved domain of DREB proteins. Different colored boxes indicated different domains

Multiple sequence alignment showed that DREB A4 and A5 have a close homologous evolutionary relationship (Fig. 2A). The conserved protein motifs obtained using the MEME program is shown (Fig. 2B). The top 10 enriched motifs were identified (Fig. S2). We compiled Major MEME motif sequences (Table S2). Noteworthily, we observed that motif 4 was present only in Group A1, suggesting that Group A1 genes evolved from the same gene. The structural characteristics of the conserved domain are shown in Fig. 2C; all family members demonstrated a common AP2 domain. The advanced structures of plant proteins are related to their biological functions and activities.

### Cis-regulatory elements in the promoters of DREB in *L. japonicus*

**Fig. 3:**
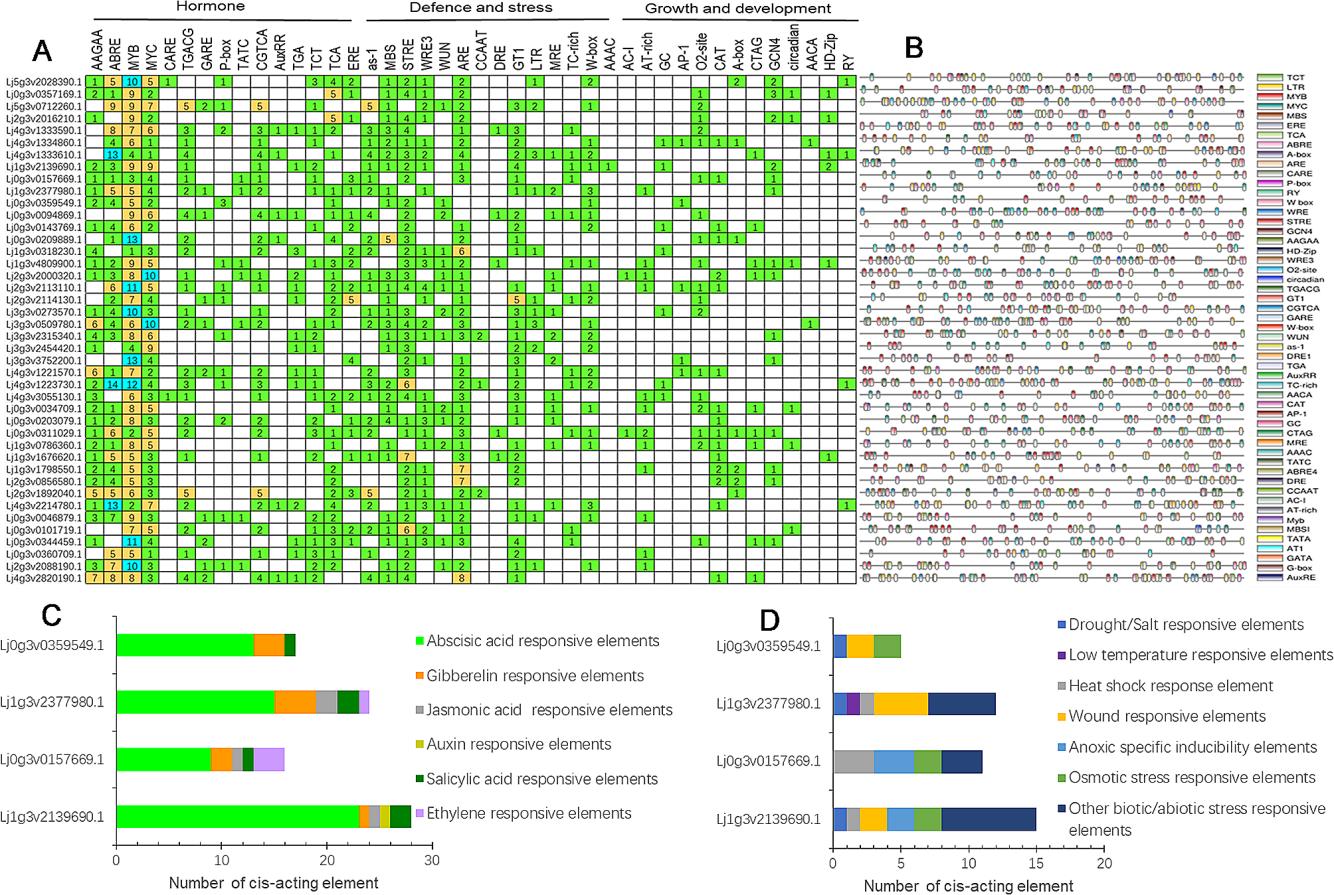
Cis-acting element analysis of DREB promoters of *Lotus japonicus*. **A** Histogram of the cis-acting elements in each DREB gene promoter. Three categories of cis-acting elements in DREB. Different numbers represent different elements. **B** Locations of cis-acting elements in the 2 kb sequences upstream of *Lotus japonicus* genes. Different types of cis-acting elements are represented by different colors. **C** Statistical analysis of cis-acting elements of the DREB A2 subfamily gene promoters related to hormone responses. **D** Statistical analysis of cis-acting elements of the DREB A2 subfamily gene promoters related to stress responses. Different cis-acting elements with similar functions are shown in the same color

The 2000 bp upstream sequence of *LjDREB* gene was extracted, and cis-regulatory elements in the promoter were obtained. In total, 42 cis-acting elements were identified and classified them into three basic categories: hormone response, stress response, and plant growth metabolic cycle (Fig. 3A, B). ABREs for abscisic acid (ABA) responsiveness are the most common elements of the DREB family. The others were associated with plant hormones, including the CGTCA- and TGACG-motif for MeJA-responsive elements, the TATC-box and P-box for gibberellin-responsive elements, and the TGA-element for auxin-responsive elements. Anaerobic induction (ARE), low-temperature responsiveness (LTR), and binding sites involved in drought inducibility (MBS and MYB) were also found. The number of cis-acting elements was also determined (Fig. 3B). We further analyzed the gene promoters of the DREB A2 group; the cis-elements were classified into hormone-related elements (Fig. 3C) and stress-related elements (Fig. 3D). Overall, the hormone and stress response genes were more abundant than the plant growth metabolic cycle genes. These results suggest that DREB genes likely play a crucial role in abiotic stress responses.

### Gene expression response patterns under drought stress

**Fig. 4:**
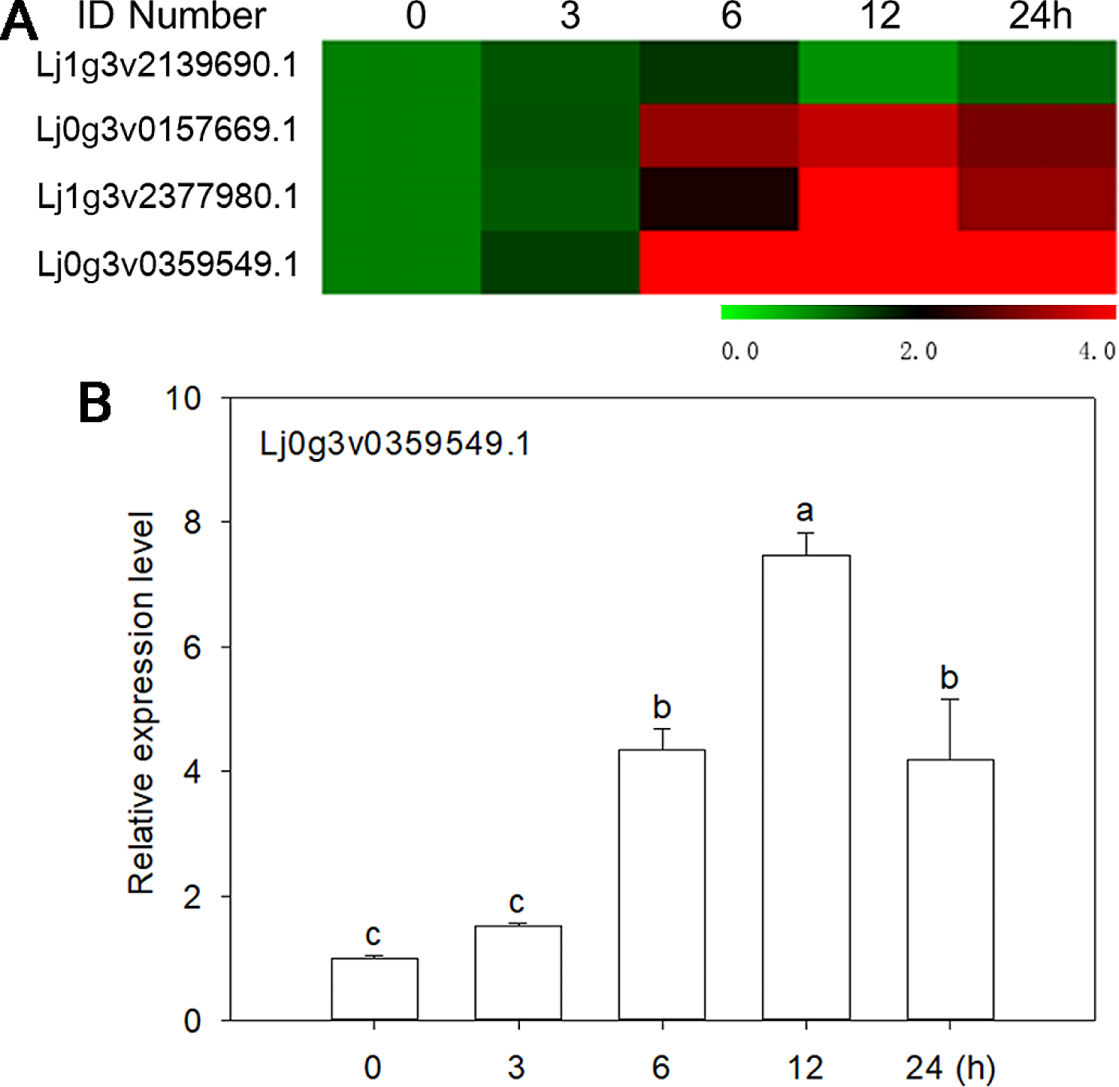
Expression levels of four DREB A2 genes under drought stress at different times. **A** Heatmap of four DREB A2 genes under drought stresses at different times. **B** Expression level of *Lj0g3v0072079.1* at different times under drought stresses

To further investigate the function of DREB A2 in the drought stress response, we identified the expression patterns of DREB A2 family genes using qRT-PCR. The results showed that the expression of three genes in this family of four genes rapidly increased, reaching the highest level at 12 h of drought stress, and then decreased (Fig. 4A). The expression level of the *Lj0g3v0359549.1* gene was the highest, reaching 7.8-fold after 12 h of drought stress (Fig. 4B). *Lj0g3v0359549.1* may play an important role in drought stress. Furthermore, *Lj0g3v0359549.1* was orthologous to AtDREB2B through sequence comparison (Fig. S3). Therefore, we named *Lj0g3v0359549.1* as *LjDREB2B*.

### Interaction network of DREB proteins

To obtain further insight into *LjDREB2B*, an interaction network was constructed using the STRING software (Fig. S4A). LjDREB2B was predicted to interact with the Delta-1-pyrroline-5-carboxylate synthase (P5CS) protein, which plays a critical role in plant drought responses. We also predicted that *LjDREB2B* interacted with Dehydration-respective protein A (RD29A), Dehydration-respective protein 22 (RD22), Heat stress transcription factor (HSFA3), and Alpha-neurotoxin homolog 7 (NHX7) which are all involved in responses to abiotic stresses. In addition, we predicted the interaction proteins of the A2 family members (Fig. S4B-D). Functional enrichment analysis was based on Gene Ontology in the network of LjDREB2B proteins that responded to salt, temperature, and abiotic stress, and water deprivation (Table S4). *LjDREB2B* proteins may play an important role in stress response. These findings are consistent with the mechanisms of *LjDREB2B* in drought response regulation.

### Overexpression of *LjDREB2B* improves drought tolerance in transgenic *Arabidopsis*

**Fig. 5:**
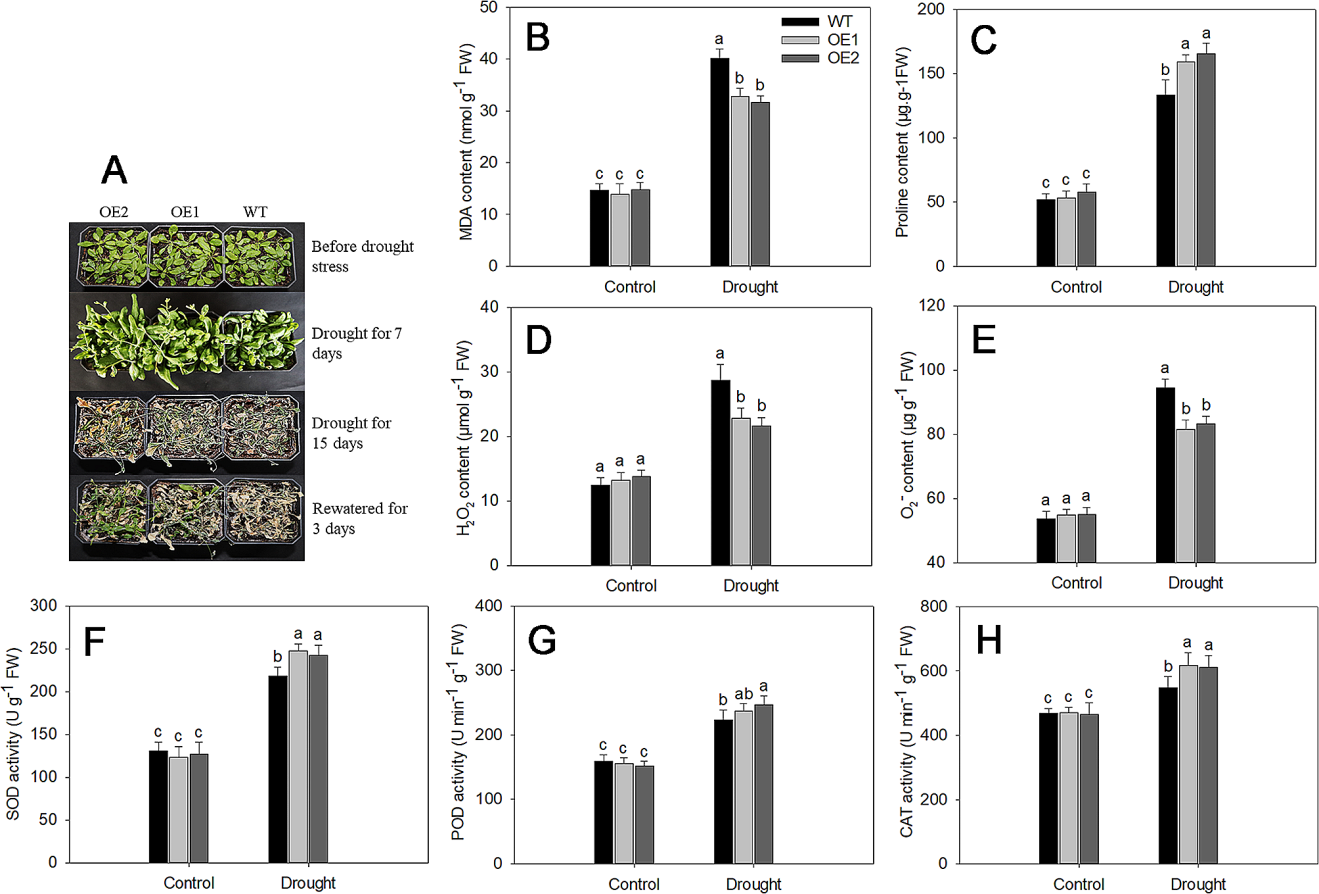
Overexpression of *LjDREB2B* in transgenic *Arabidopsis* improved drought tolerance. **A** Phenotypic observation of WT and OE transgenic *Arabidopsis* lines under drought stress. **B** MDA content. **C** Proline content. **D** SOD activity. **E** POD activity. **F** CAT activity. Different letters indicate significant differences based on the LSD test (*p* < 0.05)

**Fig. 6:**
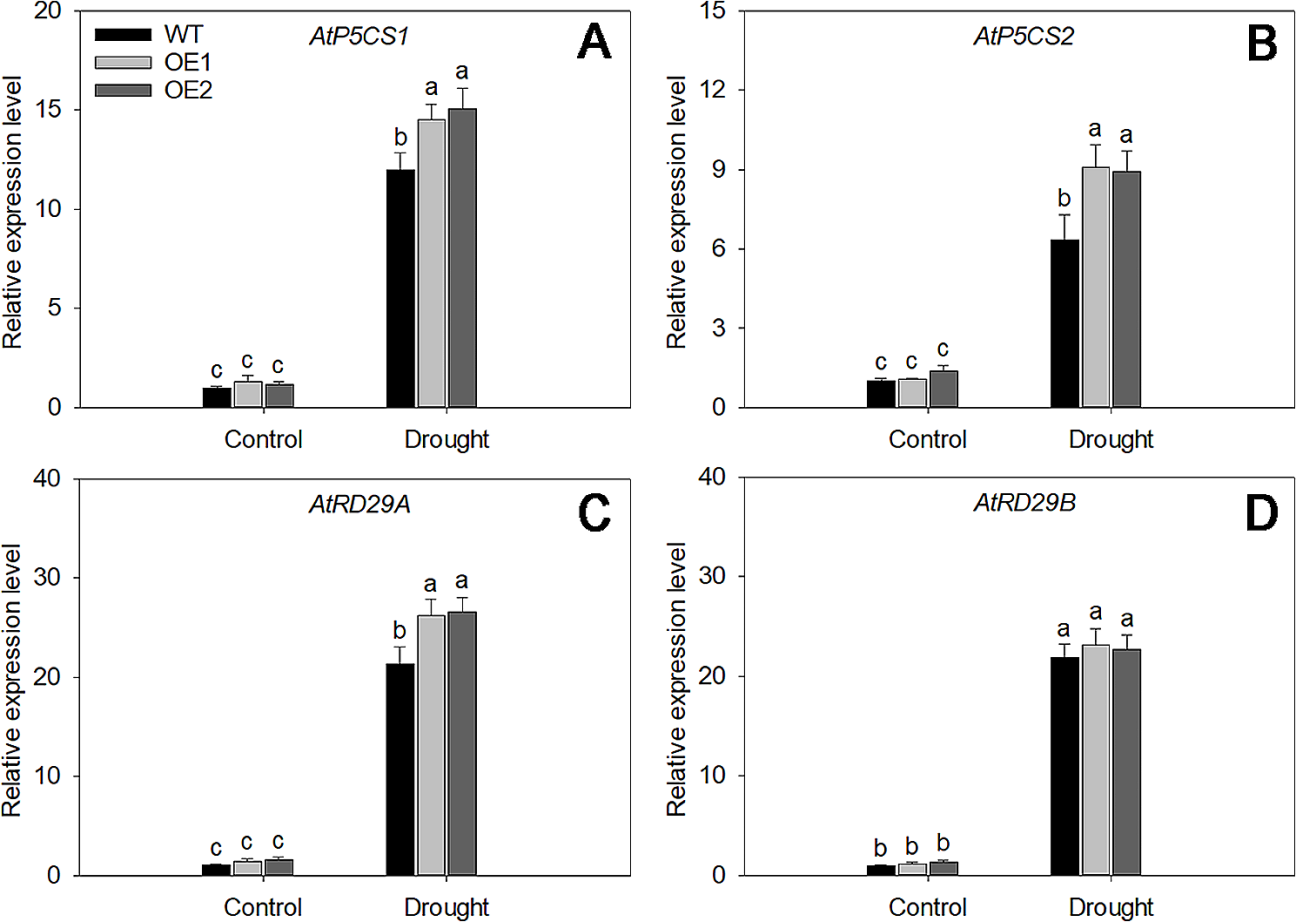
Expression levels of drought response-related genes. **A***AtP5CS1*. **B***AtP5CS2*. **C***AtRD29A*. **D***AtRD29B*. Different letters indicate significant differences based on the LSD test (*P* < 0.05)

To further investigate the function of *LjDREB2B* under drought stress, we generated 35 S::LjDREB2B overexpression transgenic *Arabidopsis* (OE) lines and selected 2 single insertion lines as drought stress (Fig. S5, Table S5). Under drought treatment, the growth of both the WT and OE lines was inhibited, although the transgenic plants showed better growth (Fig. 5A). The malondialdehyde (MDA) and proline levels of WT plants were higher and lower than those of *LjDREB2B* overexpression lines, respectively (Fig. 5B C). H_2_O_2_ and O_2_^−^ contents of WT plants were also higher than those of *LjDREB2B* overexpression lines (Fig. 5D and E). Plants have the capacity to scavenge excessive ROS by promoting an enzymatic antioxidant defense system that includes SOD, POD, and CAT. We measured the activities of SOD, POD, and CAT, and the results indicated that the activities of these enzymes rapidly increased after 7 days of drought stress (Fig. 5F-H). The enzyme activities in the OE lines were significantly higher than those in the WT lines. Furthermore, the expression levels of *AtP5CS1*, *AtP5CS2*, *AtRD29A*, and *AtRD29B* in the OE lines were significantly higher than in the WT plants after drought stress (Fig. 6A-D). Therefore, these results indicated that overexpression of *LjDREB2B* increased the drought tolerance of transgenic *Arabidopsis* plants.

## Discussion

Plant development is a complex process that occurs in various environments. DREB are an important class of TFs involved in plant development and stress responses. There are 56 DREB members divided into six subfamilies in *Arabidopsis thaliana* [31]. A total of 73 DREB members have been identified in the soybean genome [32]. In the present study, the leguminous grasses *M. truncatula* and *T. pratense* had 71 and 55 DREB members, respectively (Table S1). Forty-two DREB proteins were identified in *L. japonicus* (Fig. 1). They were distributed on branches A1–A6 of the phylogenetic tree, with the exception of A3. These results indicate that DREB family genes are widespread and evolutionarily diverse in plants. Previous studies have shown that DREB family genes have a variety of motifs and structures in plants and participate in various functions [33]. Su performed a genome-wide identification and expression analysis of DREB family genes in cotton, which indicated that the DREB family gene domains were highly conserved [34]. In the present study, the DREB family genes had different conserved motifs in *L. japonicus* (Fig. 2B and C). Some differences were observed in motif distribution among the different subfamilies, but most of the motifs were shared on the same branch. All DREB genes contain motif 1, which belongs to the conserved AP2 domain. Our results are consistent with those of previous studies [35].

Under stress, some cis-acting elements of gene promoters, such as hormone- and stress-responsive elements, recognize upstream signals and regulate downstream gene expression at the transcriptional level, thereby enabling plants to resist stress. DREB TFs are sensitive to plant hormones and play key roles in plant hormone signaling pathways [36]. Filyushins reported that AsaDREB1 promoters contain seven hormones and nine stress-responsive cis-regulatory elements [37]. Nakashima showed that most DREB genes have cis-acting AAGAA and ABRE that are responsive to ABA [38]. In the present study, the promoter elements of the DREB genes were highly diversified in *L. japonicus* (Fig. 3A and B). The most common elements were ABRE and AAGAA (Fig. 3A and B). Almost all family gene promoters have MYB/MYC and the drought-related cis-acting elements MBS, STRE, and ARE [37]. We further analyzed the gene promoters of the DREB A2 group; the cis-elements were classified into hormone- and stress-related elements (Fig. 3C, D). Therefore, DREB A2 genes may be involved in different adversity pathways and play an important role in plant drought stress.

Plant DREB TFs play important roles in transcriptional activation under abiotic stress. DREB A2 participates in drought resistance regulation in plants [39]. The expression of DREB A2 TFs was induced by drought stress [40, 41]. DREB2-type proteins are particularly important in plant responses to drought [42]. Our results show that the expression of these three DREB A2 genes was rapidly induced by drought stress (Fig. 4A). The expression level of *LjDREB2B* was the highest and reached 7.8-fold after 12 h of drought stress (Fig. 4B). DREB genes participate in hormone signaling pathways and regulate proteins interacting with downstream genes to improve plant stress tolerance [43]. We further predicted *LjDREB2B* might interact with P5CS, RD29, and RD22 (Fig. S4), although protein-protein interactions need to be further investigated. Therefore, our results indicated that *LjDREB2B* may play an important role in drought stress.

To investigate the role of *LjDREB2B* in drought stress, we overexpressed *LjDREB2B* in Arabidopsis. Under drought stress, the growth of the transgenic lines was significantly better than that of the WT lines (Fig. 5A). Previous studies have shown that *DREB* overexpression increases drought resistance [44, 45]. For instance, overexpression of *BrDREB2B* in transgenic Arabidopsis enhances tolerance to salt, heat, and drought stress [31]. Overexpression of *Phyllostachys edulis PeDREB28* improved plant salt and cold tolerance in Arabidopsis and bamboo [46]. Overexpression of *Syntrichia caninervis ScDREB10* enhanced transgenic Arabidopsis plant stress tolerance by regulating phenylpropanoid biosynthesis and starch metabolism [47]. *Eremosparton songoricum* DREB2-type transcription factor *EsDREB2B* enhances tolerance to multiple abiotic stresses in transgenic tobacco [48]. *MaDREB1F* overexpression increases banana resistance to cold and drought stress by regulating soluble sugar and proline levels and activating antioxidant systems [49]. Overexpression of *VuDREB2A* improves drought and heat stress in cowpea by enhancing osmotic adjustment, antioxidant defense, and photosynthetic efficiency [50]. In the present study, the MDA and ROS contents of the WT plants was higher than those of *LjDREB2B* overexpression lines (Fig. 5B-E). Furthermore, the proline content and antioxidant enzyme activities of the WT plants were lower than those of *LjDREB2B* overexpression lines (Fig. 5C-H). These results showed that the overexpression of *LjDREB2B* increased drought tolerance by enhancing the enzymatic antioxidant defense system in transgenic *Arabidopsis*.

Previous studies have indicated that DREB family TFs regulate the expression of downstream stress-related genes, including *HsfA3*, *RD29A*, *RD29B*, *COR15a*, *kin1*, *kin2*, and *erd10* [51, 52]. The overexpression of *DREB1A* upregulates expression of RD29A and COR15A and improves drought stress tolerance in *Arabidopsis* plants [15, 53]. Overexpression of *Ammopiptanthus nanus AnDREB5.1* increased osmotic and cold stress tolerances by enhanced antioxidant enzyme activity and expression of stress-tolerant related genes in transgenic tobacco [54]. Overexpression of *Cerasus humilis ChDREB2C* increased salt tolerance by enhancing scavenging capacity of ROS and expression of *AtSOS1*, *AtNHX1* and *AtRD29B* in transgenic plant [55]. Our results indicated that overexpression *LjDREB2B* increased the expression levels of *AtP5CS1*, *AtP5CS2*, *AtRD29A*, and *AtRD29B* (Fig. 6). These results suggest that the overexpression of *LjDREB2B* might increase drought resistance in *Arabidopsis* plants by upregulating the expression of genes related to stress. Therefore, the DREB gene family may provide high-quality plant resources and drought tolerance for molecular breeding.

## Conclusions

We performed a comprehensive analysis of the evolutionary relationships and protein features of the DREB family genes. Furthermore, the motifs and domains of the proteins and promoter cis-acting elements of the DREB genes were analyzed in *L. japonicus*. We further analyzed the expression pattern of DREB A2 in response to drought stress and found that *LjDREB2B* was strongly induced by drought stress. Additionally, our results showed that overexpression of *LjDREB*2B could increase drought resistance in transgenic *Arabidopsis* plants. Therefore, our results provide a basis for further functional studies on the DREB family genes in *L. japonicus* and resources for prospective applications in drought-resistant breeding.

## Methods

### Identification of DREB proteins

The protein sequences of the DREB family genes *Medicago truncatula*, *Trifolium pratense*, *Glycine max* and *Cajanus cajan* were downloaded from the NCBI database, and with reference to previous studies [56, 57]. The DREB protein sequences of *Arabidopsis thaliana* from the *Arabidopsis* information resource, TAIR (https://www.arabidopsis.org/) were obtained. The protein sequences of the *Lotus japonicus* were downloaded from Miyakogusa.jp 3.0 database (http://www.kazusa.or.jp/lotus/). Sequence alignment was performed using ClustalW with default parameters, considering the full-length protein sequences. The domains were checked against their respective peptide sequences in the SMART database (http://smart.embl-heidelberg.de/) and CD-Search (https://www.ncbi.nlm.nih.gov/Structure/cdd/wrpsb.cgi) [20, 21].

The isoelectric point (pI) was predicted using Compute pI/Mw software (http://www.expasy.ch/tools/pi_tool.html) and ExPASy Proteomics Server ProtParam (http://web.expasy.org/protparam/) [23].

### Constructs and plant transformation

The *LjDREB2B* gene was PCR-amplified from *Lotus japonicas* cDNA based on a gene sequence (Lj0g3v0359549.1), The *LjDREB2B* cDNA sequence was cloned into the binary pBI121vector, which carries the kanamycin resistance gene for bacterial and plant selection, and drives transgene expression using the CaMV35S promoter (Fig. S6). The construct was transformed into the Agrobacterium tumefaciens strain GV3101 by electroporation, which was then used to floral dip method in *Arabidopsis thaliana* plants. Two homozygous transgenic *Arabidopsis* lines, exhibiting high *LjDREB2B* expression levels, were chosen for subsequent drought stress experiment.

### Evolution, domain organization, and structure analysis

Phylogenetic trees was constructed with the full-length DREB proteins of *Lotus japonicus* and *Arabidopsis thaliana* were constructed using Clustal 2.1 software using with default settings to examine the evolutionary relationships, and MEGA7.0 software was used to conduct a unrooted phylogenetic analysis by the neighbor-joining (NJ) method with 1000 bootstrap replicates [31]. Conserved motifs of the DREB family of *Lotus japonicus* proteins were constructed using MEME Version 5.5.5 (http://meme-suite.org/tools/meme), top 10 most enriched motifs were selected [20, 21]. The conserved domains of the DREB family of *Lotus japonicus* were predicted using NCBI-CDD (http://www.ncbi.nlm.nih.gov/Structure/cdd/wrpsb.cgi) [20, 33]. The distribution was drawn using a visualization tool in TBtools.

### Cis‑acting element analysis

The 2000 bp sequences of the upstream DREB genes were obtained from the corresponding scaffolds (http://www.kazusa.or.jp/lotus/). The cis-acting elements of each DREB gene were predicted using PlantCARE (http://bioinformatics.psb.ugent.be/webtools/plantcare/html/) [31]. TBtools was used for drawing.

### Plant growth conditions and drought treatments

*Lotus japonicus* ecotype “MG20” was used in this study. The *Lotus japonicus* seeds were provided by Prof. Yanmin Wu’s Lab (Chinese Academy of Agricultural Sciences). *Arabidopsis thaliana* ecotype “Columbia-0” seeds were preserved in our laboratory. We selected *Lotus japonicus* seeds with full grains, planted them in culture dishes covered with double-layered filter paper, and grew them in a controlled environment with a photoperiod of 16 h/8 h (light/dark) at 24 °C and 70% relative humidity in a growth chamber. The *Lotus japonicus* seeds were planted in pots with perlite for 30 d and then subjected to drought treatment. We removed the original culture solution and then watered the solution with 20% PEG. The leaf samples were measured at 0, 3, 6, 12, and 24 h. Each treatment included three replicates in gene expression.

For the drought stress treatments, seeds of *Arabidopsis thaliana* “Columbia-0” (WT) and overexpression *LjDREB2B* transgenic *Arabidopsis thaliana* were grown in MS medium for 7 d, transplanted into nutrient soil, grown for 35 d, and not watered for 15 d, and then re-watered.

### Gene expression analysis

Total RNA was extracted from leaves using plant isolation kits (Cat. #B518631; Sangon Biotech, Shanghai, China). Total RNA was extracted and reverse transcribed, as previously described [58]. Actin2 was used as a reference gene. Table S4 lists all the specific primer sequences.

### Prediction of protein interaction network and functional annotation

The protein–protein interaction network was constructed using STRING (https://string-db.org/cgi/input.pl) [33]. Functional enrichment analysis was performed using Gene Ontology.

### Malondialdehyde content

Malondialdehyde content was determined using the thiobarbituric acid reaction method [59]. Fresh leaf samples were powdered in liquid nitrogen and homogenized in trichloroacetic acid. The supernatant was then centrifuged, extracted, and mixed with thiobarbituric acid. The mixture was boiled and centrifuged, and the absorbance was measured at 450, 532, and 600 nm.

### Proline content

The proline content was determined using the acidic ninhydrin reaction method [59]. Fresh leaf samples were powdered in liquid nitrogen, homogenized in sulfosalicylic acid, and heated in boiling water. The samples were centrifuged and the supernatant was mixed with acidic ninhydrin and glacial acetic acid. After cooling, the samples were mixed with toluene, the supernatant was collected, and the absorbance was measured at 520 nm.

### Antioxidant enzyme activities

The antioxidant enzyme activity was determined according to the method described by Nie et al. [60].

### H_2_O_2_ and O_2_^−^ contents

Fresh leaf samples (approximately 0.2 g per sample) were powdered in liquid nitrogen. H_2_O_2_ and O_2_^−^ contents were performed as described in the instruction of Suzhou Grace Biotechnology Co.,Ltd (Suzhou, Jiangsu, China).

### Statistical analysis

Statistical analyses were performed using SPSS 17.0. Mean and standard error values were calculated for variable comparisons. The experimental data were analyzed using the least significant difference (LSD) test (*P* < 0.05).

### Electronic supplementary material

Below is the link to the electronic supplementary material.

**Additional file 1: table S1.** Physical and chemical properties of DREB family proteins.

**Additional file 2: Table S2.** Sequence information for Motif 1–Motif 10.

**Additional file 3: Table S3.** Primers used in the present study.

**Additional file 4: table S4.** Functional enrichment in the network of *LjDREB2B* proteins.

**Additional file 5: Table S5.** Identification of T1 *Arabidopsis* transgenic seeds by single copy insertion.

**Additional file 6: fig. S1.** Different gene numbers for the six plant species.

**Additional file 7: fig. S2.** Detailed sequence logos of the 10 conserved motifs in *Lotus japonicus* from MEME analysis.

**Additional file 8: fig. S3.** Amino acid sequence alignment of *LjDREB2B.*

**Additional file 9: Fig. S4.** Predicted protein interaction networks of Lj0g3v0072079.1, Lj1g3v2139690.1, Lj0g3v0157669.1, and Lj1g3v2377980.1. Proteins were identified using the STRING online database with *Arabidopsis thaliana* as the background. Edges represent protein–protein associations. Nodes represent proteins, and the red nodes represent query proteins. Black, green, blue, light-sky blue, and purple lines represent co-expression, text mining, gene co-occurrence, protein homology, and experimental determination, respectively.

**Additional file 10: Fig. S5.** The expression levels of *LjDREB2B* in 4 transgenic *Arabidopsis* lines were identified by qPCR.

**Additional file 11: Fig. S6.** The vector map of overexpression *LjDREB2B* in transgenic *Arabidopsis thaliana*.

## Data Availability

The relevant data sets supporting the results of this article are included within the article and its additional files.

## Abbreviations

DREB: Dehydration responsive element binding protein
ERF: Ethylene-Responsive Element Binding Factor
TF: transcription factor
Mw: Molecular weight
pI: Isoelectric point
ABA: Abscisic acid
SA: Salicylic acid
JA: Jasmonic acid
MeJA: Methyl jasmonate
GA: Gibberellin
qRT-PCR: Quantitative real-time polymerase chain reaction
ABA: abscisic acid
MDA: malonaldehyde
P5CS: 1- pyrroline − 5- carboxylic acid synthase
NCBI: National Center for Biotechnology Information
WT: Wild-type
