# Supplementary material for: Characterization of DREB family genes in Lotus japonicus and LjDREB2B overexpression increased drought tolerance in transgenic Arabidopsis
Source: BMC Plant Biol. 2024 Jun 4;24:497. doi: 10.1186/s12870-024-05225-y (PMC11285619; doi:10.1186/s12870-024-05225-y)
Supplement: Supplementary file 2 — Additional file 2: Table S2. Sequence information for Motif 1–Motif 10. [file 12870_2024_5225_MOESM2_ESM.docx]

**Table S2. Sequence information for Motif 1–Motif 10**

| Motif | Width | Best possible match | Functional annotation |
| --- | --- | --- | --- |
| 1 | 38 | KKSRIWLGTFPTPEMAARAYDVAALALRGSSARLNFPE | DNA-binding domain found in transcription regulators in plants such as APETALA2 and EREBP (ethylene responsive element binding protein). |
| 2 | 21 | HKVYRGVRMRKWGKWVSEIRE | DNA-binding domain found in transcription regulators in plants such as APETALA2 and EREBP (ethylene responsive element binding protein). |
| 3 | 23 | LPRPASTSPRDIQAAAAKAAEMF | features not shown |
| 4 | 21 | VTLASARPKKRAGRRVFKETR | features not shown |
| 5 | 50 | QQQQHEHQQSABIASFESHJGRESSFTTTLASSSSDTPTTHEQAGATTQV | features not shown |
| 6 | 50 | MEPWYSLDDLQSTKYVDQMLSASSFYDIDSTHHLFNDVYEESDIRLWNFC | features not shown |
| 7 | 50 | DKDPQPLGTGDSLRJNALKSSVDAKAZEICNKVKKEKAEKKGVKKLNDGK | features not shown |
| 8 | 50 | EZSQTNNYGGIELAGEYGEFEDELDLLHSIDNGFYLAPPPPPYEDRIWBD | features not shown |
| 9 | 22 | DMSADSIRKKATEVGARVDALQ | features not shown |
| 10 | 49 | VMDYFEPFGVSSFEPLGSSVYLRQRDILQKFYQESRLNGSPVPTSFANP | features not shown |
