# Supplementary material for: Characterization of DREB family genes in Lotus japonicus and LjDREB2B overexpression increased drought tolerance in transgenic Arabidopsis
Source: BMC Plant Biol. 2024 Jun 4;24:497. doi: 10.1186/s12870-024-05225-y (PMC11285619; doi:10.1186/s12870-024-05225-y)
Supplement: Supplementary file 3 — Additional file 3: Table S3. Primers used in the present study. [file 12870_2024_5225_MOESM3_ESM.doc]

Table S3. Primers used in the present study

| Primer | Sequence (5' to 3') |
| --- | --- |
| LjUbi-F | CAAGGAAGGTATCCCACCG |
| LjUbi-R | TTAGAATCCACCACGAAGACG |
| Lj1g3v2139690.1-F | GATGGAAAACCAGCCTCAAA |
| Lj1g3v2139690.1-R | GAGGGAAGAGACACGATGGA |
| Lj0g3v0157669.1-F | AGAACGCTTCGTGCGAGTAT |
| Lj0g3v0157669.1-R | GTTGCAAGTGGGGAAGGTTA |
| Lj1g3v2377980.1-F | AAGTGGGTTGCTGAGATTCG |
| Lj1g3v2377980.1-R | CAGCTTTTGCAAGATCACCA |
| Lj0g3v0359549.1-F | ATAAGCCGGTTCGGAAAGTT |
| Lj0g3v0359549.1-R | ATGGCAGTGGCAAAAGTACC |
| AtActin2-F | CTAAGCTCTCAAGATCAAAGGCTTA |
| AtActin2-R | ACTAAAACGCAAAACGAAAGCGGTT |
| AtP5CS1-F | GTTTTTGAATCCCGACCTGA |
| AtP5CS1-R | TTACCCCCAACAGTCTCTGG |
| AtP5CS2-F | CAGCTTAGCCGCTCTTCTGT |
| AtP5CS2-R | AACAGGAACGCCACCATAAG |
| AtRD29A-F | GAACACTCCGGTCTCTCTGC |
| AtRD29A-R | CAATCTCCGGTACTCCTCCA |
| AtRD29B-F | AAAAGAGAGGCACCGACTCA |
| AtRD29B-R | CCGTTGACCACCGAGATAGT |
