# Supplementary material for: Characterization of DREB family genes in Lotus japonicus and LjDREB2B overexpression increased drought tolerance in transgenic Arabidopsis
Source: BMC Plant Biol. 2024 Jun 4;24:497. doi: 10.1186/s12870-024-05225-y (PMC11285619; doi:10.1186/s12870-024-05225-y)
Supplement: Supplementary file 4 — Additional file 4: table S4. Functional enrichment in the network of LjDREB2B proteins. [file 12870_2024_5225_MOESM4_ESM.docx]

**Table S4.** **Functional enrichments in the network of *LjDREB2B* interacting proteins**

| Gene Ontology | Go-term | Description | Count in network |
| --- | --- | --- | --- |
| Biological Process | GO:0009631 | Cold acclimation | 4 of 58 |
|  | GO:0010286 | Heat acclimation | 4 of 61 |
|  | GO:0010150 | Leaf senescence | 4 of 111 |
|  | GO:0009414 | Response to water deprivation | 10 of 368 |
|  | [GO:0009651](http://amigo.geneontology.org/amigo/term/GO:0009651) | Response to salt stress | 9 of 444 |
|  | GO:0009408 | Response to heat | 5 of 254 |
|  | GO:0000302 | Response to reactive oxygen species | 3 of 151 |
|  | GO:0006970 | Response to osmotic stress | 10 of 524 |
|  | GO:0009738 | Abscisic acid-activated signaling pathway | 4 of 224 |
|  | GO:0009737 | Response to abscisic acid | 9 of 540 |
|  | [GO:0010035](http://amigo.geneontology.org/amigo/term/GO:0010035) | Response to inorganic substance | 11 of 727 |
|  | GO:0045893 | Positive regulation of transcription, DNA-templated | 8 of 529 |
|  | GO:0009409 | Response to cold | 6 of 391 |
|  | [GO:0009266](http://amigo.geneontology.org/amigo/term/GO:0009266) | Response to temperature stimulus | 9 of 627 |
|  | GO:0010604 | Positive regulation of macromolecule metabolic process | 9 of 827 |
|  | GO:0048827 | Phyllome development | 5 of 471 |
|  | GO:0006979 | Response to oxidative stress | 4 of 434 |
|  | GO:1901700 | Response to oxygen-containing compound | 14 of 1582 |
|  | GO:0009628 | Response to abiotic stimulus | 16 of 2020 |
|  | GO:0048367 | Shoot system development | 6 of 829 |
|  | GO:0009725 | Response to hormone | 10 of 1543 |
|  | GO:0099402 | Plant organ development | 6 of 1030 |
|  | GO:0009755 | Hormone-mediated signaling pathway | 5 of 902 |
|  | GO:0042221 | Response to chemical | 16 of 2960 |
|  | GO:0006355 | Regulation of transcription, DNA-templated | 12 of 2386 |
|  | GO:0006950 | Response to stress | 18 of 3820 |
|  | GO:0048731 | System development | 8 of 1835 |
|  | GO:0070887 | Cellular response to chemical stimulus | 7 of 1661 |
|  | GO:0031323 | Regulation of cellular metabolic process | 13 of 3384 |
|  | [GO:0007275](http://amigo.geneontology.org/amigo/term/GO:0007275) | Multicellular organism development | 9 of 2344 |
|  | GO:0051716 | Cellular response to stimulus | 11 of 3214 |
|  | GO:0050896 | Response to stimulus | 20 of 6206 |
|  | GO:0050794 | Regulation of cellular process | 15 of 5300 |
|  | GO:0050789 | Regulation of biological process | 16 of 5971 |
| Molecular  Function | GO:0005534 | Galactose binding | 2 of 2 |
|  | GO:0000976 | Transcription cis-regulatory region binding | 7 of 954 |
|  | GO:0003700 | DNA-binding transcription factor activity | 11 of 1642 |
|  | GO:0003677 | DNA binding | 12 of 2517 |
|  | GO:0003676 | Nucleic acid binding | 13 of 4469 |
| Cellular Component | GO:0005634 | Nucleus | 14 of 6070 |
|  | GO:0043231 | Intracellular membrane-bounded organelle | 20 of 12391 |

Functional enrichments in the network originate from Gene Ontology.
