# Supplementary material for: Characterization of DREB family genes in Lotus japonicus and LjDREB2B overexpression increased drought tolerance in transgenic Arabidopsis
Source: BMC Plant Biol. 2024 Jun 4;24:497. doi: 10.1186/s12870-024-05225-y (PMC11285619; doi:10.1186/s12870-024-05225-y)
Supplement: Supplementary file 5 — Additional file 5: Table S5. Identification of T1 Arabidopsis transgenic seeds by single copy insertion. [file 12870_2024_5225_MOESM5_ESM.docx]

**Table S5. Identification of T1 Arabidopsis transgenic seeds by single copy insertion**

| Transgenic plant number | Number of positive plants | Number of negative plants | Segregation ratio | Copy number |
| --- | --- | --- | --- | --- |
| 1 | 152 | 48 | 3:1 | 1 |
| 2 | 148 | 47 | 3:1 | 1 |
| 3 | 156 | 23 | 7:1 | > 1 |
| 4 | 154 | 19 | 8:1 | > 1 |
