# Supplementary figures and images for: Characterization of DREB family genes in Lotus japonicus and LjDREB2B overexpression increased drought tolerance in transgenic Arabidopsis
Source: BMC Plant Biol. 2024 Jun 4;24:497. doi: 10.1186/s12870-024-05225-y (PMC11285619; doi:10.1186/s12870-024-05225-y)

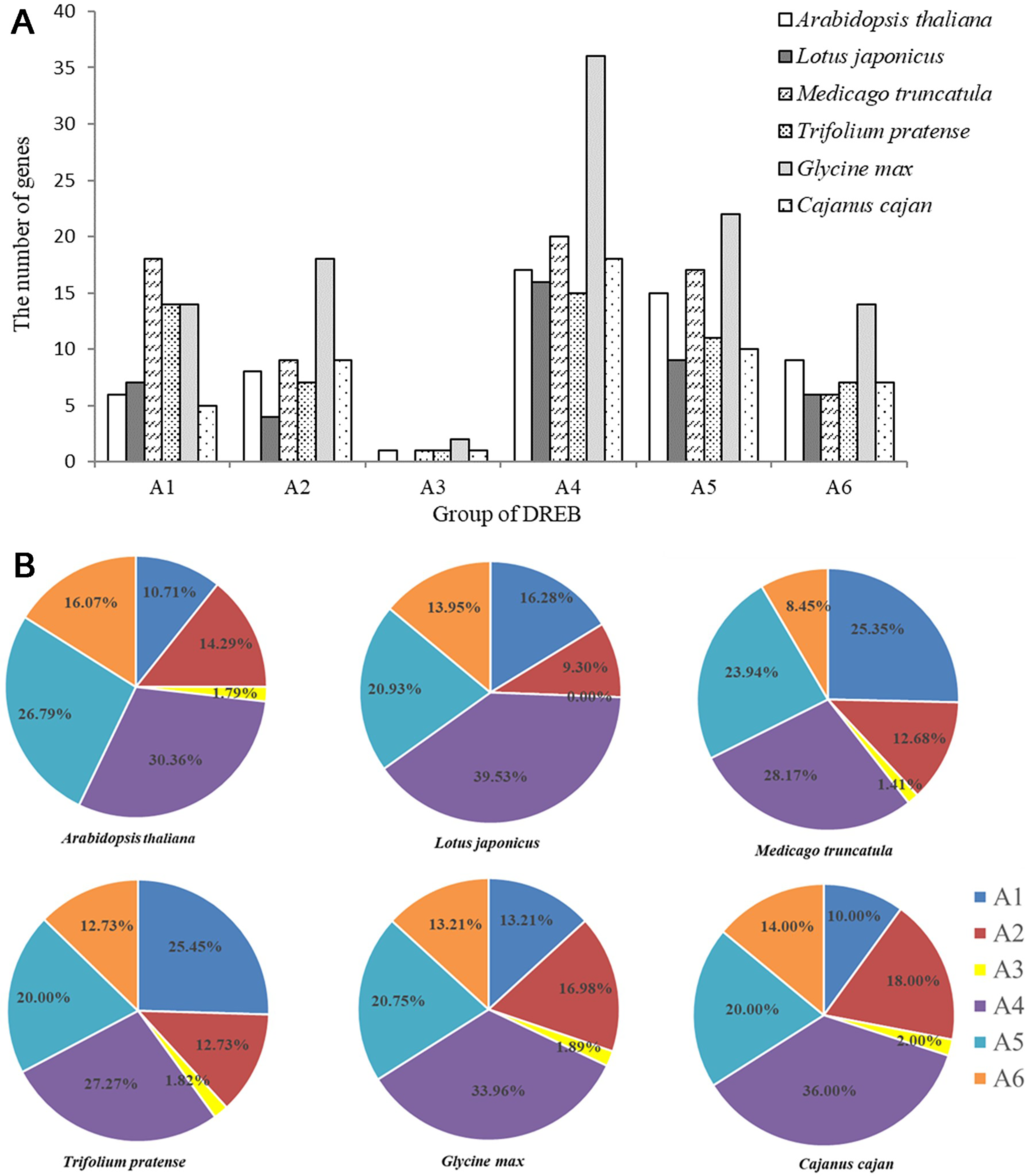

Supplement: Supplementary file 6 — Additional file 6: fig. S1. Different gene numbers for the six plant species. [file 12870_2024_5225_MOESM6_ESM.tif]
